# On assumptions and key issues in electric field modeling for ECT

**DOI:** 10.1038/s41380-024-02567-9

**Published:** 2024-04-26

**Authors:** Zhi-De Deng, Miklos Argyelan, Jeremy Miller, Thomas R. Jones, Joel Upston, Shawn M. McClintock, Christopher C. Abbott

**Affiliations:** 1 Noninvasive Neuromodulation Unit, Experimental Therapeutics and Pathophysiology Branch, National Institute of Mental Health, National Institutes of Health, Bethesda, MD USA; 2 Department of Psychiatry and Behavioral Sciences, Duke University School of Medicine, Durham, NC USA; 3 Department of Psychiatry, The Zucker Hillside Hospital, Glen Oaks, NY USA; 4 Center for Neuroscience, Feinstein Institute for Medical Research, Manhasset, NY USA; 5 Zucker School of Medicine at Hofstra/Northwell, Department of Psychiatry, Hempstead, NY USA; 6 Department of Psychiatry, University of New Mexico, Albuquerque, NM USA; 7 Division of Psychology, Department of Psychiatry, UT Southwestern Medical Center, Dallas, TX USA

**Keywords:** Depression, Neuroscience

## To the Editor:

Recently, Dr. Sartorius commented on our work [1] on assessing relationships between electric field (E-field) strength, hippocampal volume change, and electroconvulsive therapy (ECT) clinical outcomes [2]. Dr. Sartorius questioned the applicability of E-field modeling for ECT based on 1) temporal waveform dependence; 2) tissue impedance dependence; and 3) tissue anisotropy dependence. We appreciate these considerations and agree that E-field modeling would benefit from further validation and improvement. However, we must point out misconceptions of E-field modeling assumptions and provide clarification regarding some key issues.

Dr. Sartorius asserted that E-field modeling was done with the assumption of a direct current application that deviates from the alternating current waveform used in ECT, which is affected by tissue inductance and capacitance. In many models of time-dependent bioelectromagnetic phenomena, solutions to low-frequency problems commonly employ the quasi-static approximation [3, 4]. This approximation simplifies Maxwell’s equations by neglecting the wave propagation, inductive, and capacitive effects in biological tissue. The conditions for neglecting the wave propagation and inductive effects are easily satisfied due to the physical dimensions and non-magnetic nature of the tissue [3]. Tissue inductance is typically not modeled as it is related to the magnetic response of a material; in models of electrical stimulation, it is the dielectric property of the tissue that is relevant, including tissue conductivity, $\sigma$, and permittivity, $\varepsilon$. The condition for neglecting capacitive effects is that the displacement current is small compared to the conduction current, i.e., j$\omega \varepsilon /\sigma \ll 1$, where $\omega$ is the excitation frequency. For very low frequencies, e.g., 10 Hz, tissue (skin, bone, and brain) permittivity is substantial, and the capacitive effects cannot be easily ignored [5]. However, the permittivity is approximately a log-linear, decreasing function with frequency [6, 7]. For certain stimulus waveforms (e.g., monophasic square pulses with pulse width up 1 ms) used for deep brain stimulation and ECT, where the error between the electric potential calculated under the quasi-static approximation and the exact solution is limited to 5–13% [8], the capacitive effects could be ignored.

Since we assumed that the head tissues are purely resistive, the E-field is linearly proportional to the input current amplitude. Therefore, we first calculated the E-field based on an input current of 1 mA and then multiplied it by the individual treatment current (600–800 mA). Dr. Sartorius pointed out that at low current strengths such as 1 mA, the measured head impedance (so-call “static impedance”) is much higher than the impedance seen during the pulse (the “dynamic impedance”). The small-signal impedance is affected by conditions at the electrode–skin interface. To model the E-field in both low and high current situations, Unal et al. devised an impedance model of the scalp, which included a superficial scalp layer with adaptive conductivity that linearly increases with E-field up to a limit and a deep scalp layer with a fixed conductivity [9]. In their high current model, the overall scalp conductivity ranges from 0.16–0.5 S/m across four subjects. In our model, we used a scalp conductivity value of 0.465 S/m, which is within the range of appropriate scalp conductivity values to model high current ECT. Updated computational modeling pipeline for ECT has recently been proposed that accounts for dynamic changes in tissue impedance at high-current stimulation and includes data-driven scalp conductivity parameters [10].

Finally, Dr. Sartorius pointed out that strong direction-dependent effects from white matter tracts may affect the E-field distribution. Indeed, previous ECT E-field modeling investigations have incorporated white matter anisotropic conductivity [11]. Relevant to an older patient population, white matter hyperintensities may also impact E-field variability [12]. However, E-field modeling that incorporated white matter anisotropy did not improve E-field accuracy in a critical validation study with in vivo intracranial recordings in humans [13]. In another study that examined the relationship between E-field and ECT-induced brain volume expansion, we found that the incorporation of diffusion tensor imaging-derived anisotropy data to improve the E-field model produced similar regression results [14]. Nevertheless, the impact of direction-dependent effects on E-field modeling, or a generally more accurate representation of the geometry and electrical properties of various brain tissue, is an area of active and needed research.

The field of E-field modeling is grounded on working assumptions such as the quasistatic approximation to balance between complexity and practicality. The present models serve their intended purpose adequately for clinical and research applications in ECT, though refinements are possible. The future of this field will undoubtedly see more sophisticated models that will be validated against empirical data. Recent validation efforts include in vivo intracranial recordings [13, 15] and magnetic resonance current density reconstruction approaches [16]. Furthermore, ECT stimulus modeling of amplitude-determined seizure titration has been validated with in non-human primate models [17, 18] and depressed subjects [19]. E-field can be further improved with better tissue segmentations [20, 21] and conductivity values [9, 10]. Advances in E-field modeling approaches must be balanced with computational costs and complexity to achieve translational clinical impact. The context of these improvements will systematically improve the accuracy of E-field modeling. These anticipated improvements do not preclude research focused on elucidating the role of ECT E-field strength and clinical outcomes.

## References

[1] Deng Z-D, Argyelan M, Miller J, Quinn DK, Lloyd M, Jones TR, et al. Electroconvulsive therapy, electric field, neuroplasticity, and clinical outcomes. Mol Psychiatry. 2022;27:1676–82. 34853404 10.1038/s41380-021-01380-y PMC9095458

[2] Sartorius A. Electric field distribution models in ECT research. Mol Psychiatry. 2022;27:3571–2. 35304563 10.1038/s41380-022-01516-8 PMC9708590

[3] Plonsey R, Heppner D. Considerations of quasi-stationarity in electrophysiological systems. Bull Math Biophys. 1967;29:657–64. 5582145 10.1007/BF02476917

[4] Wang B, Peterchev AV, Gaugain G, Ilmoniemi RJ, Grill W, M, Bikson M et al. Quasistatic approximation in neuromodulation. arXiv. 2024. https://arxiv.org/abs/2402.00486. 10.1088/1741-2552/ad625e PMC11370654 38994790

[5] Gaugain G, Quéguiner L, Bikson M, Sauleau R, Zhadobov M, Modolo J, et al. Quasi-static approximation error of electric field analysis for transcranial current stimulation. J Neural Eng. 2023;20:016027. 10.1088/1741-2552/acb14d 36621858

[6] Gabriel S, Lau RW, Gabriel C. The dielectric properties of biological tissues: III. Parametric models for the dielectric spectrum of tissues. Phys Med Biol. 1996;41:2271–93. 8938026 10.1088/0031-9155/41/11/003

[7] Gabriel S, Lau RW, Gabriel C. The dielectric properties of biological tissues: II. Measurements in the frequency range 10 Hz to 20 GHz. Phys Med Biol. 1996;41:2251–69. 8938025 10.1088/0031-9155/41/11/002

[8] Bossetti CA, Birdno MJ, Grill WM. Analysis of the quasi-static approximation for calculating potentials generated by neural stimulation. J Neural Engg. 2008;5:44–53. 10.1088/1741-2560/5/1/005 18310810

[9] Unal G, Swami JK, Canela C, Cohen SL, Khadka N, FallahRad M, et al. Adaptive current-flow models of ECT: explaining individual static impedance, dynamic impedance, and brain current delivery. Brain Stimul. 2021;14:1154–68. 34332156 10.1016/j.brs.2021.07.012

[10] Unal G, Poon C, FallahRad M, Thahsin M, Argyelan M, Bikson M. Quasi-static pipeline in electroconvulsive therapy computational modeling. Brain Stimul. 2023;16:607–18. 36933652 10.1016/j.brs.2023.03.007 PMC10988926

[11] Lee WH, Deng Z-D, Kim T-S, Laine AF, Lisanby SH, Peterchev AV. Regional electric field induced by electroconvulsive therapy in a realistic finite element head model: influence of white matter anisotropic conductivity. Neuroimage. 2012;59:2110–23. 22032945 10.1016/j.neuroimage.2011.10.029 PMC3495594

[12] Indahlastari A, Albizu A, Boutzoukas EM, O’Shea A, Woods AJ. White matter hyperintensities affect transcranial electrical stimulation in the aging brain. Brain Stimul. 2021;14:69–73. 33217610 10.1016/j.brs.2020.11.009 PMC8174001

[13] Huang Y, Liu AA, Lafon B, Friedman D, Dayan M, Wang X, et al. Measurements and models of electric fields in the in vivo human brain during transcranial electric stimulation. eLife. 2017;6:e18834. 28169833 10.7554/eLife.18834 PMC5370189

[14] Takamiya A, Bouckaert F, Laroy M, Blommaert J, Radwan A, Khatoun A, et al. Biophysical mechanisms of electroconvulsive therapy-induced volume expansion in the medial temporal lobe: a longitudinal in vivo human imaging study. Brain Stimul. 2021;14:1038–47. 34182182 10.1016/j.brs.2021.06.011 PMC8474653

[15] Louviot S, Tyvaert L, Maillard LG, Colnat-Coulbois S, Dmochowski J, Koessler L. Transcranial Electrical Stimulation generates electric fields in deep human brain structures. Brain Stimul. 2022;15:1–12. 34742994 10.1016/j.brs.2021.11.001

[16] Eroğlu HH, Puonti O, Göksu C, Gregersen F, Siebner HR, Hanson LG, et al. On the reconstruction of magnetic resonance current density images of the human brain: pitfalls and perspectives. Neuroimage. 2021;243:118517. 34481368 10.1016/j.neuroimage.2021.118517

[17] Peterchev AV, Krystal AD, Rosa MA, Lisanby SH. Individualized low-amplitude seizure therapy: minimizing current for electroconvulsive therapy and magnetic seizure therapy. Neuropsychopharmacology. 2015;40:2076–84. 25920013 10.1038/npp.2015.122 PMC4613599

[18] Lee WH, Lisanby SH, Laine AF, Peterchev AV. Minimum electric field exposure for seizure induction with electroconvulsive therapy and magnetic seizure therapy. Neuropsychopharmacology. 2017;42:1192–1200. 27934961 10.1038/npp.2016.276 PMC5437889

[19] Abbott CC, Miller J, Farrar D, Argyelan M, Lloyd M, Squillaci T, et al. Amplitude-determined seizure-threshold, electric field modeling, and electroconvulsive therapy antidepressant and cognitive outcomes. Neuropsychopharmacology. 2023;49:640–8. 10.1038/s41386-023-01780-4 PMC10876627 38212442

[20] Puonti O, Van Leemput K, Saturnino GB, Siebner HR, Madsen KH, Thielscher A. Accurate and robust whole-head segmentation from magnetic resonance images for individualized head modeling. NeuroImage. 2020;219:117044. 32534963 10.1016/j.neuroimage.2020.117044 PMC8048089

[21] Weise K, Wartman WA, Knösche TR, Nummenmaa AR, Makarov SN. The effect of meninges on the electric fields in TES and TMS. Numerical modeling with adaptive mesh refinement. Brain Stimul. 2022;15:654–63. 35447379 10.1016/j.brs.2022.04.009

